# Estenosis Espinal de Triple Región Sintomática (EETRS): reporte de un caso y revisión narrativa de la literatura

**DOI:** 10.31053/1853.0605.v81.n1.43229

**Published:** 2024-03-27

**Authors:** Valentino Latallade, Matias Pereira Duarte, Sebastian Gamsie, Gonzalo Rodrigo Kido, Matías Gustavo Petracchi, Marcelo Fernando Gruenberg

**Affiliations:** 1 Sector Patología de Raquis del Adulto, Hospital Italiano de Buenos Aires, Argentina Buenos Aires Argentina

**Keywords:** estenosis espinal de triple región sintomática, estenosis espinal en tándem, estenosis cervical, dorsal y lumbar, symptomatic triple-region spinal stenosis, tandem spinal stenosis, cervical, thoracic, and lumbar stenosis, estenose espinhal de tríplice região sintomática, estenose espinhal em tandem, estenose cervical, dorsal e lombar

## Abstract

**Introducción:**

La estenosis espinal de las tres regiones de la columna en simultáneo es una entidad infrecuente, que requiere una adecuada valoración clínica e imagenológica. Actualmente no existen guías establecidas para su abordaje diagnóstico y terapéutico.

**Objetivo:**

Describir, partiendo del reporte de un caso, la presentación clínica, el tratamiento y la evolución en un paciente con triple estenosis y contrastarlo con la evidencia disponible a través de una revisión narrativa de la literatura.

**Presentación del caso:**

Mujer de 69 años de edad que consultó con un cuadro de paraparesia progresiva asociado a ciatalgia derecha y signos de motoneurona superior positivos. Imagenologicamente se constató una triple estenosis: cervical, dorsal y lumbar, realizándose una descompresión y resección tumoral dorsal asociado al tratamiento conservador de las estenosis cervical y lumbar, con una evolución favorable al año postquirúrgico.

**Conclusión:**

La estenosis espinal de triple región sintomática es una condición rara, las valoraciones clínicas y radiológicas adecuadas permitirán un diagnóstico correcto con un abordaje adecuado y oportuno.

## Abstract (en)

**Objective:**

Describe, based on a case report, the clinical presentation, treatment, and progression in a patient with triple stenosis, and to compare it with available evidence through a narrative literature review.

**Case Presentation:**

A 69-year-old woman presented with progressive paraparesis associated with right sciatica and positive signs of upper motor neuron involvement. Imaging confirmed triple stenosis: cervical, thoracic, and lumbar. She underwent dorsal decompression and tumor resection, with conservative treatment for cervical and lumbar stenoses, showing favorable progress one year post-surgery.

**Conclusion:**

Symptomatic triple-region spinal stenosis is an uncommon condition. Proper clinical and radiological assessments will enable an accurate diagnosis with appropriate and timely intervention.

## Resumo

**Objetivo:**

Descrever, a partir do relato de um caso, a apresentação clínica, o tratamento e a evolução em um paciente com estenose tripla e compará-lo com as evidências disponíveis em uma revisão narrativa da literatura.

**Apresentação do caso:**

Uma mulher de 69 anos procurou atendimento com um quadro de paraparesia progressiva associada à ciatalgia direita e sinais positivos de neurônio motor superior. Radiologicamente, foi constatada uma estenose tripla: cervical, dorsal e lombar. Foi realizada uma descompressão e ressecção tumoral dorsal, juntamente com tratamento conservador para as estenoses cervical e lombar, com uma evolução favorável um ano após a cirurgia.

**Conclusão:**

A estenose espinhal de três regiões sintomáticas é uma condição rara. Avaliações clínicas e radiológicas apropriadas permitirão um diagnóstico correto com abordagem adequada e oportuna.

## Introducción

La estenosis espinal en tándem (EET) es definida como el estrechamiento sincrónico del diámetro del canal espinal en al menos dos regiones distintas de la columna vertebral, con una prevalencia imagenológica de entre 0.2% al 11% de la población general. ^1^ - ^2^

Clínicamente se presenta con síntomas asociados de motoneurona superior e inferior. Dagi y cols. ^3^ describen una tríada típica caracterizada por 1) claudicación y pérdida de fuerza de miembros inferiores; 2) alteración de la marcha con pérdida de la estabilidad; y 3) signos de motoneurona superior como hiperreflexia, clonus y Babinski positivos.

La evidencia disponible respecto al abordaje quirúrgico de la EET es escasa, siendo la casuística aún más limitada cuando la estenosis se presenta en tres segmentos sincrónicamente (cervical, torácico y lumbar), denominándose estenosis espinal de triple región sintomática (EETRS). Su baja frecuencia asociada a la diversidad de signos y síntomas posibles generan un cuadro sumamente complejo, cuyo diagnóstico y resolución quirúrgica no han sido adecuadamente definidos, presentando múltiples opciones terapéuticas (descompresión de región única o múltiple, por etapas o simultánea). ^4^

El objetivo de este trabajo es describir, partiendo del reporte de un caso, la presentación clínica, el tratamiento y la evolución en un paciente con triple estenosis y contrastarlo con la evidencia disponible a través de una revisión narrativa de la literatura.

## Materiales y Métodos

Previa aprobación por el Comité de ética de nuestra Institución se realizó una búsqueda bibliográfica en la base de datos de PUBMED utilizando los siguientes términos: "Estenosis espinal en tándem", "Estenosis en tándem" o "Estenosis cervical, dorsal y lumbar". Incluimos todos los reportes de caso, casos y controles, estudios de cohortes y revisiones que evalúan pacientes adultos con EET y EETRS, donde se evalúen los métodos diagnósticos y tratamientos propuestos, con un seguimiento mínimo de 1 año. Los artículos que no discutían enfermedades espinales, que incluían pacientes pediátricos o que incluían patología de una sola región fueron excluidos. Presentamos el caso de una paciente con estenosis en tándem en los 3 segmentos de la columna vertebral. Describimos su presentación clínica, su evaluación imagenológica, su tratamiento y los resultados a los 12 meses de seguimiento comparándolos con los resultados de nuestra búsqueda bibliográfica.

## Resultados

A partir de nuestra búsqueda bibliográfica 84 artículos fueron identificados, 54 artículos fueron excluidos basados en título y resumen. De los 30 artículos restantes, 11 fueron excluidos luego de la lectura del texto completo, quedando 19 artículos destinados a la elaboración de esta revisión narrativa. (Fig. I)

**Figura I:**
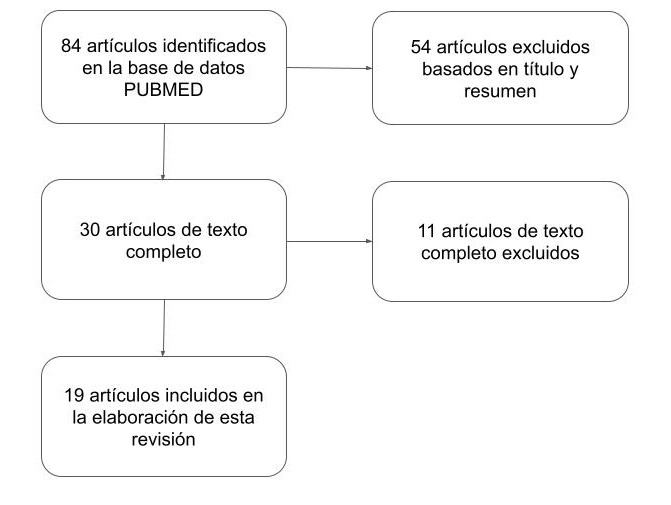
Diagrama de flujo de la búsqueda bibliográfica.

### Presentación de caso

Una paciente de sexo femenino de 69 años de edad, consulta en la central de emergencias por un cuadro de paraparesia progresiva e incapacitante (ASIA C, Nurik 5) de 1 año de evolución asociado a ciatalgia derecha e incontinencia de esfínteres subaguda.Al examen físico se constata una paraparesia con fuerza motora M3/5 en ambos miembros inferiores de L2 a S1, con hiperreflexia rotuliana y aquilianabilateral, signos de Hoffman positivos en ambos miembros superiores, clonus y Babinski positivos en miembros inferiores asociado a dolor ciático derecho de intensidad 9/10 según la escala visual análoga (EVA) en el territorio de la raíz L5 y maniobra de Lassegue y Bragard positivas. Los escores de mielopatía cervical fueron: Escala japonesa modificada ^5^ (mJOA) 9/17, escala de Nurick ^6^ 5.

### Estudios por imágenes

Una Resonancia Magnética Nuclear (RMN) realizada de urgencia reveló tres puntos de estenosis del canal raquimedular: 1) un canal estrecho cervical degenerativo C5-C6 con mielomalacia focal; 2) una tumoración intrarraquídea intradural extramedularD6-D7 con impronta en la médula espinal de 15 mm x 8 mm x 7 mm; y 3) una extrusión discal posterolateral derecha L4-L5. (Fig. II)

**Figura II:**
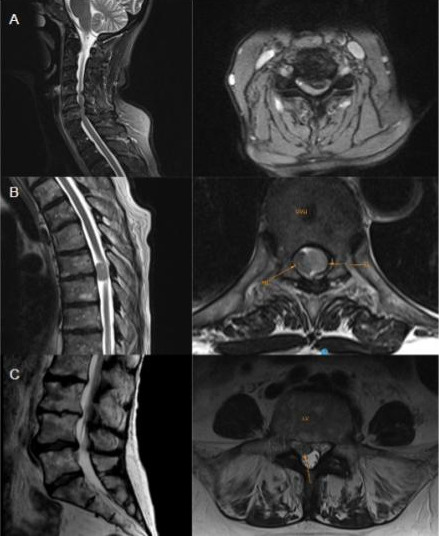
Paciente femenina de 69 años de edad. Resonancia magnética de columna sin contraste en donde se evidencia: A) Corte sagital y axial de columna cervical que evidencia una estenosis C5-C6 posterolateral izquierda debida a una protrusión discal con signos de mielomalacia B) Corte sagital y axial en D6-D7 con tumoración intradural-extramedular C) Corte sagital y axial lumbar con extrusión discal posterolateral derecha L4-L5.

#### Tratamiento quirúrgico y evolución postoperatoria

Confirmado el diagnóstico de EETRS, se decidió realizar una descompresión D6-D7 por vía posterior mediante laminectomía y exéresis del tumor intradural extramedular dorsal, asociado a bloqueo intraquirúrgico derecho L4-L5. (Fig. III) A nivel cervical, la estenosis ya era conocida y no se evidencio un empeoramiento del cuadro clínico principalmente a nivel de los miembros superiores. Razón por la cual, se atribuyó la rápida progresión de los síntomas al cuadro oncológico recientemente diagnosticado. La cirugía fue realizada bajo monitoreo intraoperatorio de potenciales evocados somatosensitivos y motores. El tiempo operatorio total fue de 2 horas y 20 minutos, bajo anestesia general. El análisis anatomopatológico fue consistente con meningioma transicional WHO grado I. ^7^ Durante el periodo postoperatorio inmediato, la paciente evolucionó favorablemente, presentando una remisión de ciatalgia con una EVA 3/10. El alta hospitalaria fue al tercer día postoperatorio, logrando marcha con andador, y con diuresis espontánea efectiva. En los controles de los siete y catorce días postoperatorios se evidenció una notable mejoría de fuerza motora con dolor ciático de 2/10, logrando deambular con bastón al mes, y sin él a los tres meses postoperatorios. El último control al año postoperatorio evidenció un dolor residual de 1/10, con un déficit M4 para L5 derecho, sin inestabilidad en la marcha y signos de motoneurona superior disminuidos, mJOA 17/17, Nurick 1. La paciente continuó con la presencia del signo de Hoffman bilateral, sin embargo, el signo de Lhermitte persistió negativo y nunca evidenció la pérdida de la destreza motora fina en miembros superiores.

**Figura III:**
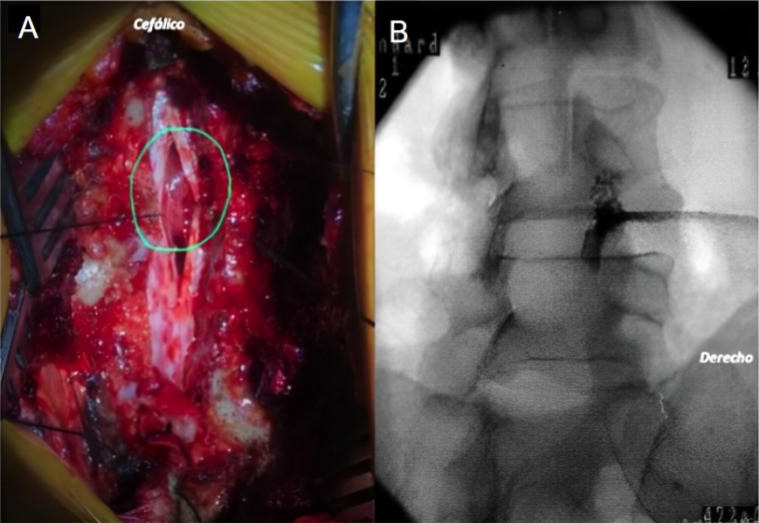
Paciente femenina de 69 años de edad. A) Imagen intraquirúrgica que evidencia la apertura de duramadre con tumoración dorsal intradural extramedular, a través de una laminectomía D6-D7 central B) Imagen fluoroscópica intraquirúrgica de bloqueo perirradicular L4-L5 derecho.

## Discusión y/o Conclusión

Este trabajo presenta el caso de una paciente que consulta con una paraparesia espástica progresiva asociada a ciatalgia derecha y diagnóstico de EETRS, la cual fue intervenida quirúrgicamente de una de las lesiones y se realizó tratamiento conservador de las otras dos con buena evolución postoperatoria y remisión casi completa de su sintomatología. La EET fue reportada por primera vez en 1957 por *Brain y cols. ^8^ ,* quienes describieron un paciente con estenosis espinal cérvico-lumbar. Sin embargo, el término de estenosis espinal en tándem propiamente dicho fue introducido por *Dagi y cols. ^3^* en 1987 para describir la estenosis espinal sintomática concurrente a nivel cervical y lumbar. En 2016 *Uehara y cols.* ^9^ clasificaron la EET en cuatro subtipos según la región: cervicotorácica, toracolumbar, cérvico-toraco-lumbar y cérvico-lumbar. Nuestro caso se trató de una EET cérvico-toraco-lumbar, caso infrecuente ya que la mayoría de los reportes en la literatura abordan la estenosis en tándem con compresión en dos niveles únicamente.Las manifestaciones clínicas de la EET son sumamente variables dependiendo de la localización de las áreas estenóticas y de la severidad de las mismas. *LeBan y cols.* ^10^ y *Kenneth y cols.* ^11^ reportan la dificultad de realizar un diagnóstico correcto debido a la presentación clínica combinada de síntomas de motoneurona superior e inferior. Estos autores reportan que los síntomas de un nivelpueden predominar sobre los síntomas de otro nivel o bien enmascararlos, por lo que podría omitirse la posibilidad de la presencia de un segundo o tercer nivel patológico. *Bhandutia y cols.* ^12^ describen en su serie de 33 pacientes una incidencia de 45% de diagnóstico tardío, con graves consecuencias en la evolución de los mismos, por lo que recomiendan considerar al EET como diagnóstico diferencial para pacientes con síntomas de estenosis espinal. En cuanto al tratamiento, *Dagi y cols.* ^3^ recomiendan la descompresión de ambos niveles, cervical y lumbar, sugiriendo comenzar por el más sintomático. *Esklander y cols.* ^13^ , en su estudio comparativo de 43 pacientes, contraponen la descompresión simultánea versus en etapas sin encontrar diferencias significativas en cuanto a la tasa de complicaciones y los resultados funcionales postoperatorios. Sin embargo, ellos aconsejan la descompresión por etapas basándose en la disminución de las complicaciones asociadas a la pérdida sanguínea. *Epstein y cols.* ^14^ siguiendo la premisa de *Dagi y cols.* ^3^ , concluyeron que el orden correcto para el tratamiento quirúrgico en EET dependía de la gravedad de la mielopatía y la radiculopatía. En su serie, la descompresión cervical mejoró los síntomas radiculares lumbares. De manera similar, en nuestro caso, la descompresión dorsal pudo haber sido favorable para la mejoría sintomática asociada a bloqueo perirradicular lumbar. Intuimos que la estenosis cervical, al ser un proceso degenerativo crónico, podría haber encontrado mecanismos de adaptación que le permitieron no manifestarse clínicamente, pero que el tumor, de velocidad de crecimiento más elevado, habría sido responsable de prácticamente la totalidad de los síntomas de motoneurona superior. En línea con lo descrito, *Luo y cols.* ^15^ realizaron una revisión retrospectiva de 47 pacientes con EET sometidos a descompresión por etapas clasificándolos en dos grupos (cervical o lumbar) según la primera región intervenida. Ellos reportan que los pacientes que se sometieron primero a una cirugía cervical no requirieron de una segunda etapa, mientras que los del grupo lumbar presentaron una exacerbación de los síntomas en relación a la estenosis cervical. Todo lo precedente refuerza lo propuesto por *Epstein y cols*. ^14^ , quienes concluyen que en presencia de EET cérvico-lumbar, la región cervical debería ser intervenida primeramente en pacientes sintomáticos. La mayoría de los reportes en la literatura abordan la EET con compresión en los niveles cervical y lumbar. Encontramos pocos reportes de casos de EET que incluyen la región dorsal, y ninguno con un tumor asociado. La mayoría son publicaciones con estenosis cervicodorsales o dorsolumbares por osificaciones del ligamento longitudinal común posterior. *Chen y cols.* ^16^ y *Hu y cols.* ^17^ reportaron revisiones retrospectivas de 15 y 16 casos respectivamente, de osificación en tándem cérvico-torácicas. Ambos reportan una resolución quirúrgica utilizando una única incisión posterior con mejoras significativas en las puntuaciones mJOA y de Nurick a los 6 meses de la cirugía, pero a expensas de una alta tasa de complicaciones intraoperatorias y postoperatorias (>50%, 8/15 pacientes) que incluyeron fuga de líquido cefalorraquídeo (LCR), hematomas de herida, parálisis de C5 y deterioro neurológico. Estos reportes sugieren utilizar una estrategia quirúrgica agresiva con precaución y recalcando la buena comunicación preoperatoria con los pacientes. Finalmente, *Schaffer y cols.* ^18^ reportaron un caso de EETRS tratado con cirugía simultánea de las tres regiones, advirtiendo que esta puede asociarse a una prolongación de la estadía hospitalaria. Mientras que *Jannelli y cols.* ^19^ presentaron un caso de EETRS a quien se realizó una descompresión por etapas, primero cervical, luego lumbar y por último dorsal con una mejoría final de su paraparesia que se retrasó dos años y medio debido al retraso del diagnóstico de la patología dorsal. En este último caso, realizaron inicialmente solo imágenes de columna cervical, disintiendo del nuestro, en donde ante la disociación clínico imagenológica del cuadro, se consideró de vital importancia efectuar la resonancia magnética del raquis completo. El examen físico junto a las imágenes fueron la clave para resolver de manera temprana el desafío diagnóstico y terapéutico que representa la triple estenosis sintomática. Contrariamente a lo que se describe en la literatura, en nuestro caso se comenzó por la región dorsal debido a que se trataba de un tumor, que causaba una estenosis severa compatible con los síntomas de la paciente. Se obtuvo un alta hospitalaria precoz y una muy buena evolución postoperatoria, con mejoría significativa de su cuadro de déficit neurológico. Este artículo reporta una revisión narrativa de la literatura referente a la triple estenosis raquídea sintomática en tandem a partir de un caso problema. Las limitaciones de este trabajo están principalmente relacionadas a la baja incidencia en el reporte de esta patología, ya que la casi totalidad de la bibliografía incluida son referentes a reporte de casos y pequeñas series de pacientes, constituyendo un nivel de evidencia bajo. Adicionalmente, se evidencia una gran heterogeneidad en la modalidad de reporte de esos casos y series de pacientes, haciendo énfasis, cada publicación, en un aspecto diferente de tema, sea el diagnóstico, el abordaje, el tratamiento quirúrgico o la evolución postoperatoria. A pesar de todo esto, ésta es, según nuestro conocimiento, la primera revisión narrativa realizada sobre este tema y creemos haber desarrollado de manera armoniosa y amena a la lectura cada uno de los aspectos inherentes a esta rara entidad.A modo de conclusión, presentamos un caso de una paciente con EETRS con compromiso neurológico progresivo, que requirió descompresión dorsal, mostrando evolución favorable. Si bien la presencia de estenosis múltiple es una condición rara, su sospecha diagnóstica a través de una correcta valoración clínica permite un tratamiento y control oportuno. La EETRS debe considerarse dentro de los diagnósticos diferenciales para pacientes con signos y síntomas de estenosis espinal y de motoneurona superior e inferior. Se requieren nuevos estudios de mejor calidad, para poder establecer guías diagnósticas y terapéuticas adecuadas en estos casos.
